# Synthesis and Optoelectronic Properties of Perylene Diimide-Based Liquid Crystals

**DOI:** 10.3390/molecules30040799

**Published:** 2025-02-09

**Authors:** Shiyi Qiao, Ruijuan Liao, Mingsi Xie, Xiaoli Song, Ao Zhang, Yi Fang, Chunxiu Zhang, Haifeng Yu

**Affiliations:** 1 School of Printing and Packaging Engineering, Beijing Institute of Graphic Communication, Beijing 102600, China; 2 Key Laboratory of Polymer Chemistry and Physics of Ministry of Education, School of Materials Science and Engineering, Peking University, Beijing 100871, China

**Keywords:** perylene diimide, liquid crystals, π-π stacking, charge carrier mobility, aggregation-induced emission

## Abstract

Perylene diimide (PDI), initially synthesized and explored as an organic dye, has since gained significant recognition for its outstanding optical and electronic properties. Early research primarily focused on its vibrant coloration; however, the resolution of solubility challenges has revealed its broader potential. PDIs exhibit exceptional optical characteristics, including strong absorption and high fluorescence quantum yield, along with remarkable electronic properties, such as high electron affinity and superior charge carrier mobility. Furthermore, the robust π-π stacking interactions and liquid crystalline behavior of PDIs facilitate precise their self-assembly into highly ordered structures, positioning them as valuable materials for advanced applications in optoelectronics, photonics, and nanotechnology. This article provides a comprehensive review of the progress made in the design, synthesis, and optoelectronic performance of PDI-based liquid crystals. It explores how various substituents and their placement on the PDI core impact the properties of these liquid crystal molecules and discusses the challenges and opportunities that shape this rapidly evolving class of optical materials. This review is strictly focused on PDIs and does not cover their elongated or laterally extended derivatives, nor does it include monoimide or ester compounds.

## 1. Introduction

As early as 1913, PDIs were first synthesized by Kardos and gained widespread recognition as a lightfast vat dye [1]. Imide groups, known for their high resonance energy and structural stability in aromatic heterocycles, combine with perylene to form PDIs, resulting in a highly persistent and robust molecular structure [2]. The typical synthesis of PDIs begins with the oxidation of acenaphthene to form naphthalene-1,4,5,8-tetracarboxylic dianhydride. This intermediate is then converted into an imide by reaction with ammonia, followed by dimerization with molten alkali to yield PDIs. Additionally, PDIs can be saponified with hot concentrated sulfuric acid to produce perylene tetracarboxylic dianhydride (PTCDA) [3].

Due to the poor solubility of PDIs and their derivatives, these compounds initially garnered limited attention and were primarily used as dyes. It was not until 1959 that Geissler and Remy first reported the fluorescence behavior of perylene dyes in solution, marking a significant milestone in the exploration of their optical properties [4]. However, the low solubility of perylene dyes significantly hindered the study and practical application of their fluorescence properties. To address this challenge, Heinz Langhals proposed modifying the imide positions of PDIs by substituting them with soluble alkyl or aryl groups, thereby enhancing the solubility of PDI derivatives and facilitating the further exploration of their potential [5]. In the 1990s, Langhals reported that the introduction of tert-butyl-modified phenyl groups at the amine positions of PDIs significantly enhanced their solubility and dissolution rate in organic solvents. This improvement was attributed to the steric hindrance caused by the bulky substituents, which pushed them away from the planar chromophore, thereby reducing the face-to-face π-stacking interactions between PDI molecules. Among various substituents, tert-butyl groups were found to be more effective at increasing solubility compared to primary or secondary alkyl groups. Long-chain secondary alkyl groups also exhibited better solubilizing effects than long-chain primary alkyl groups, the latter of which sometimes reduced solubility. For cycloalkyl substituents, solubility was found to vary with ring size. PDIs substituted with medium-sized cycloalkyl groups exhibited the lowest solubility, while those with cyclotetradecyl groups showed the highest solubility [6,7,8].

Once the solubility challenges of PDI derivatives were addressed, researchers began to explore their broader potential and applications. The rigid, large, planar aromatic conjugated system of PDIs and their derivatives, which closely aligns with the molecular structure of discotic liquid crystals, sparked significant interest in their liquid crystalline properties. This unique structural feature led to extensive studies on their ability to form well-ordered mesophases, further expanding their applicability in advanced material design and optoelectronic technologies [9]. In 1993, Klaus Müllen and his team made a groundbreaking contribution by reporting the first liquid crystalline derivatives of the perylene core. They achieved this by incorporating urazole units and four flexible alkyl chains into the perylene structure, resulting in perylene derivatives that exhibited discotic liquid crystal mesophases. Additionally, by varying the number and length of the flexible alkyl chains, they were able to induce a structural transition from a columnar mesophase to a lamellar phase, demonstrating the tunability of these materials’ mesomorphic behavior [10]. The liquid crystalline behavior of PDI derivatives was first discovered in 1997 by Gregg and his colleagues, who reported a series of PDI derivatives featuring linear or branched aliphatic side chains. This pioneering work highlighted the potential of these materials to exhibit liquid crystalline phases, opening new avenues for the exploration of their mesomorphic properties and applications [11]. Subsequently, Würthner conducted extensive research on PDIs, focusing particularly on their synthesis, functionalization, and self-assembly behavior. His work has significantly advanced the understanding of PDI derivatives, shedding light on their structural versatility and potential for a wide range of applications in optoelectronics and materials science [12,13,14,15,16]. Following this, researchers began to conduct extensive studies and explore novel PDI-based liquid crystal materials, motivated by their distinctive properties and potential for groundbreaking applications across various fields, including optoelectronics, photonics, and materials science [17,18,19].

This review is organized according to the various substitution positions and types of substituents on PDIs. It focuses on the molecular design of PDI-based liquid crystal materials, exploring how different substituents influence their optoelectronic properties, while also addressing the challenges and opportunities associated with this emerging class of materials [20,21].

## 2. Design and Synthesis of PDI Derivatives

**Figure 1:**
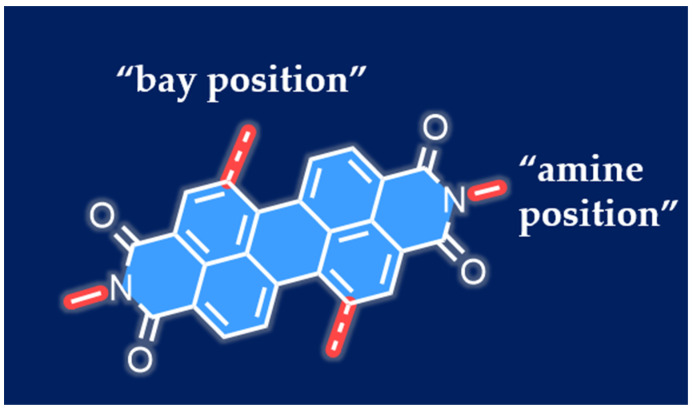
Schematic diagram of the molecular structure of PDIs. The blue part represents the core of the perylene diimide molecule, while the red part indicates the common substitution positions of perylene diimide.

Modifying the substituents of PDIs can significantly influence their chemical and physical properties [9]. As shown in Figure 1, typically, the substituents on the imide nitrogen positions of the PDI molecular structure are referred to as the “amine positions”, while those on the 1, 6, 7, and 12 positions of the perylene core are known as the “bay positions.” The substituents at the amine positions have minimal impact on the electronic and optical properties at the molecular level but can influence aggregation, solubility, and other structural characteristics [22]. In contrast, the “bay positions” play a significant role in determining the electronic and optical properties of PDIs. As a typical n-type organic semiconductor, PDIs exhibit near-unity fluorescence quantum yield, high photochemical stability, and strong electron-accepting capabilities. These attributes render PDIs highly suitable for a wide range of emerging applications in optoelectronics and related fields [23]. To date, PDIs and their related monoimide derivatives have been extensively utilized in a range of electronic and optical applications, such as organic field-effect transistors (OFETs) [24,25,26,27,28], fluorescent solar concentrators [29,30], electrophotographic devices [31,32], dye lasers, organic photovoltaic cells (OPVs) [33], and optical power limiting [34,35].

### 2.1. PDI Derivatives Modified at the Amine Position

The modification of the imide position in PDIs involves the amidation of anhydrides with amino-containing substituents, such as long-chain or branched alkylamines, alkoxyamines, anilines, and methylamines [36].

**Figure 2:**
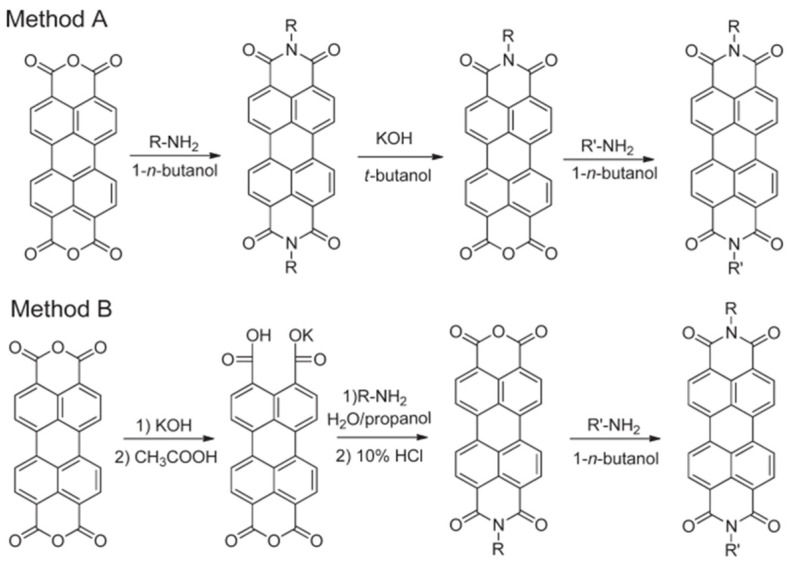
Two methods for preparing non-symmetrical PDIs with Distinct substituents at each imide position [36].

This process enhances the solubility of PDIs while simultaneously tuning its optoelectronic properties and self-assembly behavior. Given that PDIs possess two symmetrical imide positions, there are two modification approaches in Figure 2: symmetrical substitution and non-symmetrical substitution [37,38]. Symmetrical substitution is typically achieved by reacting PTCDA with aliphatic or aromatic amines as reactants [6,39,40]. This reaction is carried out through a condensation process in high-boiling-point solvents, such as imidazole, porphyrins, or N-methyl-2-pyrrolidone (NMP), and typically in the presence of a metal catalyst, often zinc acetate [41]. This method typically yields high efficiency. Non-symmetrical substitution can be achieved through two primary approaches. The first approach involves the partial hydrolysis of symmetrical PDIs to produce perylene monoimide–monoanhydride compounds, with a yield of approximately 50%. These compounds are then subjected to amidation reactions with different amines, leading to the formation of non-symmetrically substituted PDIs [33]. The second approach involves the partial hydrolysis of PTCDA to form a mixed anhydride-dicarboxylate intermediate, which is then subjected to sequential imidation reactions to yield non-symmetrically substituted PDIs. In comparison, the first method generally offers higher yields and simpler purification, making it the more commonly employed approach [36,42,43,44,45].

#### 2.1.1. PDI Derivatives with Symmetrical Substitution at the Amine Position

**Figure 3:**
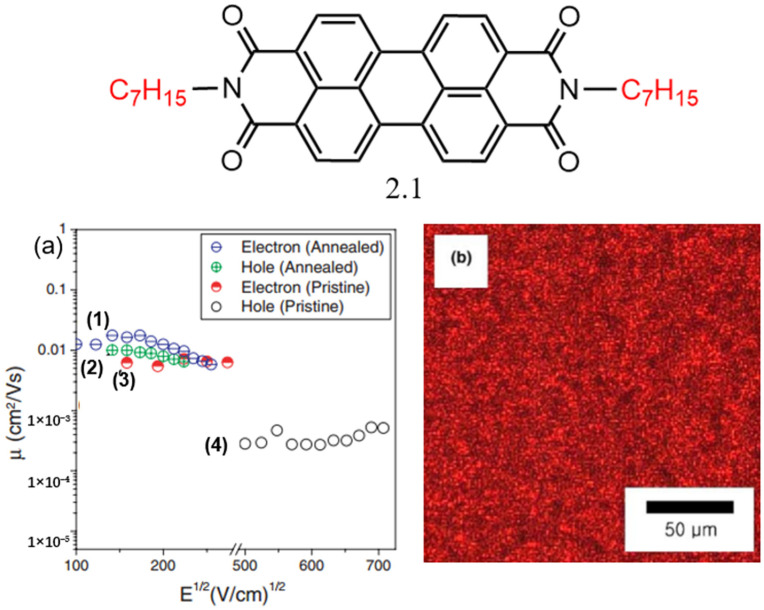
Molecular structure of PDI derivative. (**a**) Charge mobility of PDI derivative **2.1** as a function of electric field: electron mobility (1) and hole mobility (2) for annealed **2.1** films, electron mobility (3) and hole mobility (4) for pristine **2.1** films; (**b**) polarizing microscope image of PDI derivative **2.1** [46].

Kim et al. reported a PDI derivative (**2.1**) with the symmetrical substitution of alkyl chains at the amine positions as illustrated in Figure 3 [46]. Generally, dialkyl-substituted PDIs exhibit lower lowest unoccupied molecular orbital (LUMO) energy levels, which enhances their effectiveness as electron acceptors compared to other aromatic PDIs with similar bandgaps [47]. This opens up opportunities for efficient charge separation at the interface between electronically complementary aromatic PDIs. PDI derivative **2.1** demonstrated excellent liquid crystal (LC) properties. In devices with the original thin films, relatively low hole mobility and higher electron mobility were observed. However, upon annealing the organic thin film in its LC phase, a more organized structure was formed, leading to a significant increase in hole mobility to 0.00851 cm^2^/(V·s) and a slight increase in electron mobility to 0.01191 cm^2^/(V·s). ITO/PDI derivative **2.1**/Al devices were fabricated, and the drift mobilities of electrons and holes were evaluated using the time-of-flight (TOF) technique. Under an electric field of 186 (V/cm)^1^/^2^, the electron and hole mobilities were measured to be 0.0139 cm^2^/(V·s) and 0.0089 cm^2^/(V·s), respectively, with negative charge carriers (electrons) exhibiting faster mobility than positive ones.

**Figure 4:**
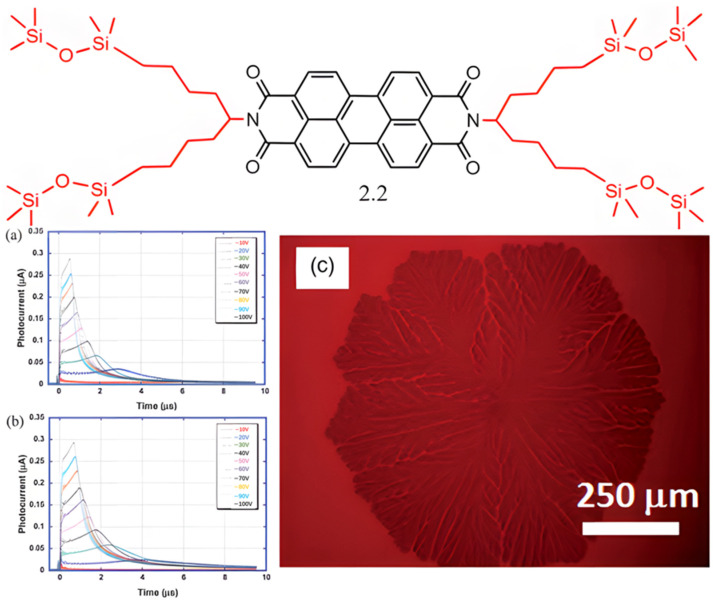
Molecular structure of PDI derivative **2.2**: (**a**) transient photocurrent curve at 30 °C; (**b**) transient photocurrent curve at 70 °C; (**c**) POM image of PDI derivative **2.2** [48].

Akinari Sonoda and colleagues synthesized a liquid crystalline semiconductor, designated as compound **2.2**, based on PDIs featuring a 1,1,1,3,3-pentamethyldisiloxane chain as depicted in Figure 4 [48]. The incorporation of oligosiloxane groups, owing to the increased rotational flexibility of the Si–O bond, imparts greater molecular flexibility compared to alkyl groups. This structural feature significantly improves the solubility of molecules with oligosiloxane chains in a variety of solvents. Additionally, it reduces the crystallinity of the mesogenic units, lowers the crystallization temperature, and consequently narrows the LC temperature range [15,16]. Moreover, the oligosiloxane chains promote the formation of a one-dimensional columnar structure. Differential scanning calorimetry (DSC) and polarized optical microscopy (POM) analyses confirmed that PDI derivative **2.2** exhibits an ordered columnar phase that remains stable at room temperature. TOF measurements showed that the electron mobility of compound **2.2** in this ordered columnar phase at room temperature exceeds 0.1 cm^2^ V^−1^ s^−1^. This high electron mobility is attributed to the efficient molecular packing within the columnar stacks. Remarkably, the electron transport properties remained consistent during phase transitions between low-temperature and high-temperature columnar phases, with minimal variation in electron mobility. The mobility values were reproducible and stable across different sample sizes, and the photocurrent signal remained stable even after storage in ambient air. Additionally, PDI derivative **2.2** is soluble in a broad range of organic solvents, excluding alcohols, and can form thin films of columnar aggregates aligned parallel to the substrate through spin-coating. Notably, uniaxially aligned LC films with columnar stacks can be prepared using a rubbing transfer method, showcasing the compound’s potential for practical applications.

**Figure 5:**
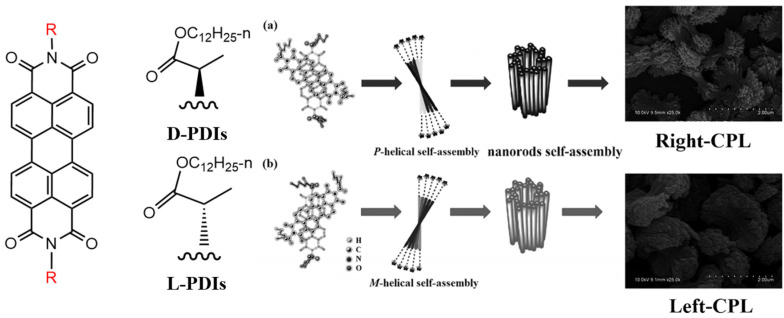
Molecular structures of D-PDIs and L-PDIs: (**a**) schematic illustration of the potential self-assembly mechanism of D-PDIs; (**b**) schematic illustration of the potential self-assembly mechanism of L-PDIs [49].

Cheng et al. successfully designed and synthesized two enantiomeric PDI derivatives incorporating D-alanine and L-alanine, referred to as D-PDIs and L-PDIs, respectively [49]. As presented in Figure 5, perylene is inherently a fluorescent-emitting chromophore, and by introducing chiral amino acid units at the amine positions of perylene, Cheng et al. developed circularly polarized luminescence (CPL)-emissive materials. Under UV light excitation (λex = 365 nm), the D/L-PDI derivatives that dissolved in chloroform (a good solvent) exhibited bright yellow fluorescence with emission peaks at 531 nm and 578 nm, achieving a high fluorescence quantum yield of 0.67. Upon the addition of methanol (a poor solvent), the fluorescence intensity decreased significantly, likely due to the aggregation-caused quenching (ACQ) effect. However, when the methanol fraction (fm) reached 95%, a new fluorescence peak emerged at approximately 643 nm, attributed to the supramolecular self-assembly of the D/L-PDIs via intermolecular π-π interactions. Meanwhile, at 578 nm, the fluorescence lifetime of D/L-PDIs exhibited single-exponential decay with a lifetime of 5.48 ns. At 643 nm (15.91 ns, 75%), the fluorescence lifetime increased significantly, attributed to aggregation at fm = 99%. Although no circular dichroism (CD) or CPL signals were observed for the D/L-PDI enantiomers in chloroform, prominent mirror-image Cotton effects and CPL emissions were detected in the aggregated state. SEM revealed that samples prepared from chloroform solutions formed D/L-PDI particles with diameters of approximately 300 nm. Notably, L-PDIs assembled into an m-helical stacking structure, whereas D-PDIs adopted a p-helical stacking structure, attributed to the steric effects of the ester groups from the chiral amino acid enantiomers. The optical anisotropy factor (glum) for the D/L-PDIs reached as high as 0.02 at fm = 99%, representing exceptionally strong CPL emission compared to most reported organic CPL materials. This pronounced CPL performance was attributed to the intermolecular π-π interaction-driven self-assembly in the aggregated state.

**Figure 6:**
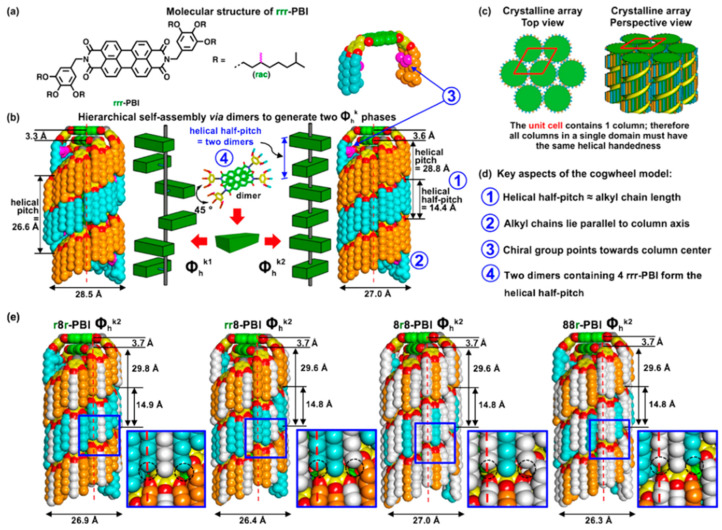
A cogwheel model of self-organization. (**a**) The molecular structure of rrr-PBI where r stands for racemic dm8*. The chiral methyl group is indicated in pink. (**b**) The two crystalline columnar hexagonal (Φ_h_^k^) phases are generated via the hierarchical self-organization of PBI dimers rotated around the column axis being offset from the column axis in Φ_h_^k1^ and centered on the column axis in Φ_h_^k2^. Due to its columnar shape and the absence of alkyl groups perpendicular or tilted to the long axes of the column, the intercolumnar interdigitation of alkyl chains is absent, and therefore, we denote Φ_h_^k2^ as the “cogwheel” assembly. (**c**) The formation of a Φ_h_^k^ array with only one helical column in the unit cell drives helical deracemization between columns. (**d**) Key aspects of the cogwheel model. (**e**) Supramolecular columns assembled from r8r-, rr8-,8r8-, and88r-PBI, where 8 represents the n-octyl group, and insets illustrate the incomplete space filling by the alkyl ends from the 3 and 5 positions of the dendron. Dashed circles in insets indicate empty space on the column periphery [50].

Percec et al. designed and synthesized a series of perylene bisimide (PBI) molecules with sequence-defined dendritic structures, enabling the formation of helical columnar supramolecular self-assemblies as demonstrated in Figure 6 [50]. They systematically examined the influence of alkyl chains of varying lengths (C_n_H_2n+1_, n = 6, 7, 9, 10) on self-assembly kinetics and structural stability. The study revealed that the 9r9 sequence—a precisely arranged dendritic architecture—enabled the formation of highly ordered helical columnar crystals at unprecedented heating and cooling rates of up to 50 °C/min, making it the fastest crystallizing supramolecular or covalent macromolecule reported to date. This remarkable behavior was attributed to the optimized tertiary structure and dynamic flexibility of the alkyl chains, which facilitate rapid molecular self-organization into highly ordered assemblies. To elucidate the structural and phase transition behavior, advanced characterization techniques such as fiber X-ray diffraction (XRD), DSC, and solid-state nuclear magnetic resonance (NMR) were employed. The findings demonstrated that the molecular packing of 9r9-PBI offers enhanced space-filling efficiency and significantly faster self-assembly compared to other PBI derivatives. Building on these insights, the authors proposed a novel supramolecular self-assembly mechanism based on the cogwheel model, offering a framework for achieving high structural order using achiral building blocks. These results pave the way for developing advanced optoelectronic materials with rapid response characteristics, such as liquid crystal displays and other high-performance soft materials.

**Figure 7:**
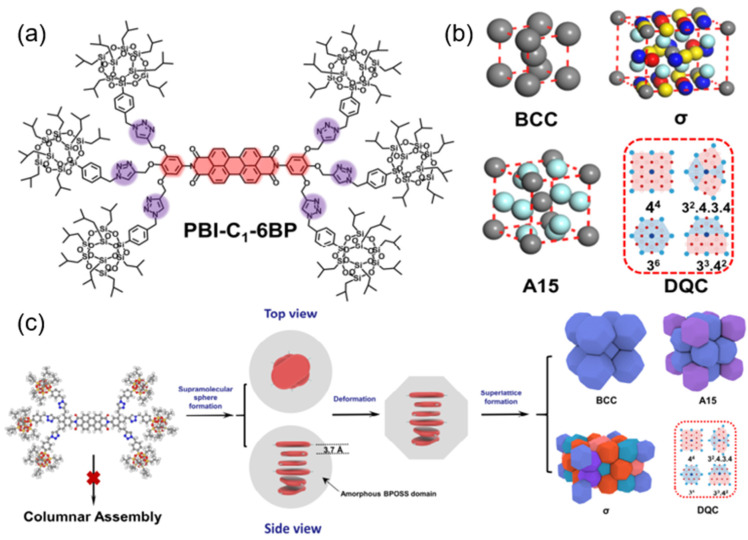
(**a**) The molecular structure of PBI-C1-6BP (The red part represents the core of the perylene diimide molecule, while the purple part corresponds to the 1,2,3-triazole segment). (**b**) Packing models of the A15 phase, σ phase, BCC phase, and the two-dimensional tiling pattern of the DQC phase are illustrated. (**c**) A schematic representation of the hierarchical self-assembly mechanism of POSS-based PBIs, where the central red blocks denote perylene cores and the gray shell of the supramolecular spheres represents amorphous BPOSS cages [51].

Huang et al. reported the design and synthesis of a series of chemically symmetric PBI molecules, functionalized with polyhedral oligomeric silsesquioxane (POSS) cages at their periphery as represented in Figure 7 [51]. Unlike conventional PBIs, which predominantly self-assemble into columnar or lamellar structures driven by strong π-π stacking interactions, the incorporation of bulky POSS cages introduces significant steric hindrance, resulting in the formation of unprecedented spherical supramolecular structures, including A15, σ, dodecagonal quasicrystalline (DQC), and body-centered cubic (BCC) phases. The steric repulsion from the rigid POSS units disrupts typical columnar packing, facilitating the emergence of these highly ordered spherical assemblies. Notably, the study uncovered an atypical inverse phase transition from the BCC to the σ phase with increasing annealing temperature, providing valuable insights into the thermodynamic stability and phase behavior of PBI-based supramolecular systems. These findings expand the functional versatility of PBI-based materials and open new avenues for their application in advanced optoelectronic devices and high-performance soft materials.

#### 2.1.2. PDI Derivatives with Non-Symmetric Substitution at the Amine Position

**Figure 8:**
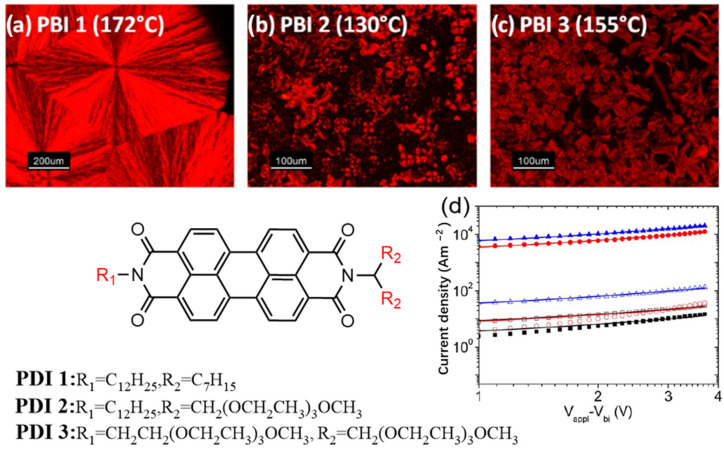
Molecular structures of PDIs 1-3: (**a**) Crystalline phase of PDIs 1 at 172 °C. (**b**) Liquid crystalline phase of PDI 2 at 130 °C. (**c**) Liquid crystalline phase of PDI 3 at 155 °C. (**d**) Current density–voltage (J–V) characteristics of electronic devices, showing the performance of PDI 1 (black squares), PDI 2 (red circles), and PDI 3 (blue triangles) before (empty symbols) and after annealing (filled symbols) [52].

Mukundan et al. reported three different PDI derivatives, where the nature of the substituents varied from hydrophobic alkyl chains to hydrophilic oligoethylene glycol (OEG) chains as outlined in Figure 8 [52]. By modifying the properties of the substituents, they investigated their impact on molecular self-assembly, structural order, and electronic transport properties. POM and DSC analyses revealed that PDI derivative 1 exhibited a three-dimensional crystalline structure, while derivatives 2 and 3 formed a two-dimensional liquid crystalline phase. After annealing, the liquid crystalline materials exhibited enhanced electronic transport efficiency due to their self-healing properties. At room temperature, the electronic mobility of these derivatives was low in their unannealed state (10^−5^ cm^2^/V·s). However, following annealing, the electronic mobility of the liquid crystalline PDIs 2 and 3 significantly increased, reaching 10^−3^ cm^2^/V·s, while the mobility of crystalline PDI 1 decreased. AFM imaging revealed that the annealed PDI 2 films exhibited smooth, step-like structures, indicating highly ordered molecular stacking, which facilitated electron conduction. These findings suggest that liquid crystalline ordering plays a key role in enhancing charge transport in organic materials.

### 2.2. Both the Amine Position and the Bay Position Are Modified in the PDI Derivative

**Figure 9:**
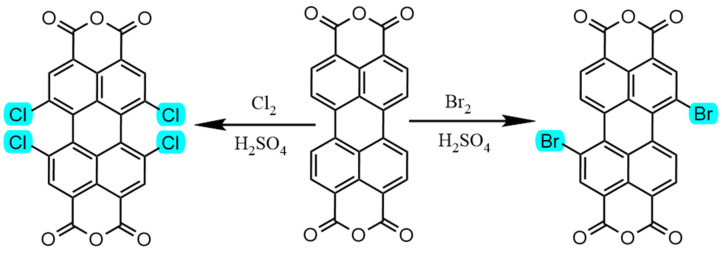
Intermediates of two PDI derivatives with bay region modification (The highlighted part represents the halogen substituents that are easy to modify).

Bay region modification involves introducing various substituents at the bay positions (i.e., positions 1, 6, 7, and 12) of the perylene core. Modifying the bay positions is more challenging than altering the imide positions because the amine positions often need to be modified first to improve solubility. This enhanced solubility is crucial for the successful attachment of substituent groups at the bay positions [53,54,55,56]. As indicated in Figure 9, PDI derivatives with bay region modifications are typically synthesized by first preparing the corresponding intermediates through processes such as halogenation, nitration, and oxidation, followed by further reactions. The synthesis usually begins with 3,4,9,10-perylene tetracarboxylic dianhydride as the precursor. Iodine is employed as a catalyst, and the reaction with **2.2 **equivalents of bromine in concentrated sulfuric acid results in the formation of the major product, 1,7-dibromo-3,4,9,10-perylene tetracarboxylic dianhydride [36,57]. The product is then subjected to amidation to enhance its solubility, transforming it into 1,7-dibromo-3,4,9,10-PDIs. The bromine atom at the bay position of 1,7-dibromo-3,4,9,10-PDIs is highly reactive and can be easily replaced by various nucleophiles [24,58]. Compared to PDI molecules with unmodified bay positions, the introduction of functional groups at the bay region significantly alters their photophysical properties and solubility [59]. This effect is influenced by factors such as the substitution method, the size and volume of the substituents, the number of substituents, and the electronic properties of the substituent groups [60,61,62,63]. Bay region modification allows for the adjustment of the highest occupied molecular orbital (HOMO) and LUMO energy levels of the compounds, thereby modifying their optoelectronic properties [64,65,66]. On the other hand, bulky substituents at the bay region are pushed out of the plane of the PDI derivatives due to steric interactions [67]. This can cause the distortion of the two naphthalene subunits in the PDI derivative, disrupting the π-π face-to-face stacking and, in turn, significantly increasing solubility by several orders of magnitude [68]. Substituents introduced at the bay region also play a crucial role in altering the electronic configuration of the perylene core [69,70]. By introducing electron-donating or electron-withdrawing groups at the bay positions, the light absorption and emission properties of PDI derivatives can be tuned across a broad spectrum, ranging from the ultraviolet to the near-infrared regions [71,72]. Due to the intramolecular charge transfer (ICT) effect, the introduction of electron-donating substituents such as pyrrolidine, piperidine, and alkyl primary amines at the bay positions causes the maximum fluorescence emission peak of the corresponding PDI derivatives to red-shift into the near-infrared region [9,63,73,74].

**Figure 10:**
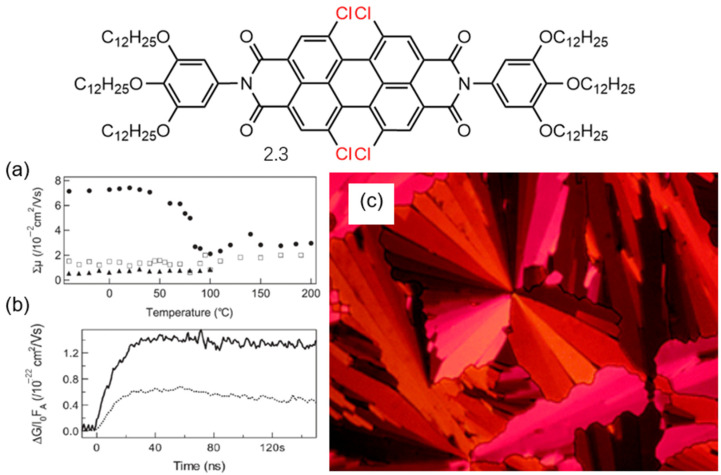
Molecular structure of PDI derivative **2.3**: (**a**) Sum of minimum isotropic charge carrier mobilities as determined by PR-TRMC for PDI derivative **2.3** (circular). (square and triangular shapes represent the test results of the precursors of PDI derivative **2.3**). (**b**) Photoconductive transients obtained from FP-TRMC measurements of 60:40 molar ratio (perylene–HBC) thin films at 500 nm, with approximately 1 × 10^15^ absorbed photons, for PDI derivative **2.3** (solid line) blended with HBC-PhC12 (dotted line represent the test results of the precursors of PDI derivative **2.3**). (**c**) Optical liquid crystal textures at crossed polarizers of PDI derivative **2.3** cooled from the isotropic phase [23].

Würthner et al. reported a PDI derivative, **2.3**, featuring four chlorine substituents at the bay region as described in Figure 10 [23]. The presence of chlorine substituents stabilizes the Colh phase of the compound, indicating that PDI derivative **2.3** exhibits a uniform π-π stacking distance along the stacking axis. This ordered π-π arrangement facilitates electron hopping, enabling the Col phase to exhibit high charge carrier mobility, similar to that of non-chlorinated compounds. When mixed with hexa-peri-hexabenzocoronene (HBC) derivatives, PDI derivative **2.3** also demonstrated enhanced carrier lifetimes, suggesting that chlorinated derivatives may play a key role in devices requiring extended carrier lifetimes. However, compared to the parent compound, PDI derivative **2.3** exhibits a narrower phase width, reduced transparency, and a higher melting point. Overall, the results indicate that chlorinated PDI derivatives have the potential to enhance charge carrier mobility and prolong carrier lifetimes.

The self-assembly of PDIs with NH groups and large bay substituents originates from the self-assembly behavior of π-conjugated rod-like molecules containing p-terphenyl groups at their hydrogen bonding termini, which is significantly influenced by the presence of bulky side chains [75].

**Figure 11:**
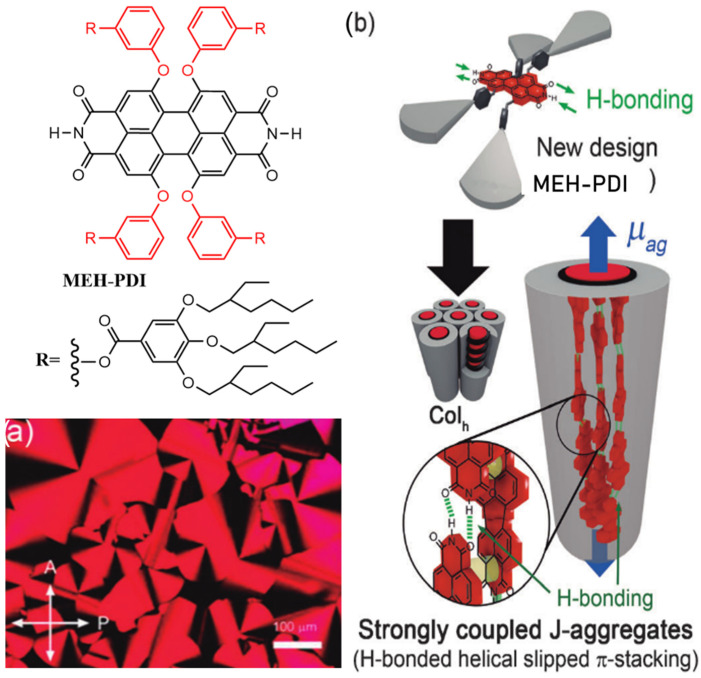
Molecular structure of MEH-PDIs (The black part represents the core structure of the perylene diimide molecule, while the red part corresponds to the substituent groups): (**a**) POM image of MEH-PDIs at 230 °C. (**b**) Schematic representation of MEH-PDIs assembled into a columnar hexagonal LC phase, highlighting the orthogonal orientation of the PBI units. (The blue arrows indicate the direction of the main transition dipole moment (mag) of the PDI molecules in MEH-PDIs) [76].

Würthner et al. designed MEH-PBI, which features N-H groups at the imide positions and is functionalized at the bay positions with four dendritic wedges, each carrying branched 2-ethylhexyl groups as highlighted in Figure 11 [76]. The UV/Vis spectra of MEH-PDIs in MCH solution and spin-coated films exhibit a sharp, intense band with a maximum at 621 nm. Compared to the monomeric PDIs (λ_max_ = 529 nm in MCH), this high-intensity band shows a red-shift, indicating the formation of J-aggregates in both solution and bulk states. POM images of MEH-PDIs reveal pseudo-focal conic textures, suggesting the formation of a columnar LC phase. DSC studies show a single LC phase within the temperature range of −47 °C to 263 °C. A uniform alignment of the columnar phase of MEH-PDIs was successfully achieved using mechanical shearing, solution shearing, and friction transfer, resulting in oriented LC phases with the columnar axes parallel to the surface and shear direction. The PDI cores are arranged with their transition dipole moments parallel to the columnar axis, representing an unprecedented structural organization in π-conjugated columnar liquid crystals. Small- and wide-angle X-ray diffraction analyses revealed a helical structure, with three self-assembled, hydrogen-bonded PDI chains forming individual columns of the columnar hexagonal phase. This remarkable columnar LC assembly mode offers potential applications in novel anisotropic liquid crystalline materials for photonics. Furthermore, the strong coupling of these molecular oscillators within the one-dimensional J-aggregates, along with their ability to align into highly anisotropic films, holds significant promise for optical applications.

**Figure 12:**
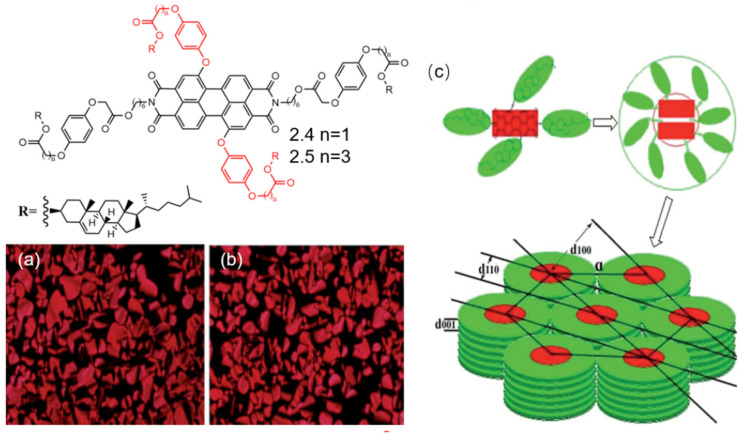
Molecular structures of PDI derivatives **2.4** and **2.5** (The black part represents the core structure of the perylene diimide molecule, while the red part corresponds to the substituent groups): (**a**) POM image of PDI derivative **2.4**, showing the textures on cooling at 130 °C (×400); (**b**) POM image of PDI derivative **2.5**, displaying the textures on cooling at 130 °C (×400); (**c**) schematic illustration of the hexagonal columnar phase stacking for both derivatives [77].

Wang et al. synthesized two PDI derivatives, **2.4** and **2.5**, with cholesterol groups attached at both the amine and bay positions as evidenced in Figure 12 [77]. The POM and DSC results demonstrated that both PDI derivatives exhibited LC phases with relatively low mesomorphic temperatures and broad LC phase ranges. In particular, the mesomorphic temperature of PDI derivative **2.5** was as low as 32 °C, with a mesophase range spanning an impressive 202 °C, which is rare for perylene liquid crystals. The influence of different spacer groups led to a lower mesomorphic temperature and a broader mesophase range in PDI derivative **2.5** compared to PDI derivative **2.4**, suggesting that longer spacer groups favor the formation of more stable mesophases. This highlights the superior mesomorphic properties of cholesterol-substituted PDI liquid crystals over alkyl-substituted perylene liquid crystals. The incorporation of cholesterol groups contributed to lower intermediate phase temperatures and wider phase transition temperature ranges. XRD analysis revealed that both derivatives exhibited hexagonal columnar phases, and the liquid crystalline alkyl chains might adopt coiled, folded, or interdigitated configurations. In terms of luminescence performance, the large alkyl units in the cholesterol structure restricted the rotation of the phenyl groups, minimizing energy loss and enhancing fluorescence intensity. These restricted intramolecular rotation mechanisms are commonly used to explain fluorescence enhancement in the field of aggregation-induced emission. Additionally, the increased number of alkyl units led to greater steric hindrance, reducing the formation of ordered aggregates in solution and further boosting fluorescence intensity. Moreover, PDI derivative **2.5** demonstrated stronger fluorescence than PDI derivative **2.4**, indicating that longer spacer groups are beneficial for fluorescence. Notably, the fluorescence quantum yield of PDI derivative **2.5** reached an exceptional 0.96, making it one of the most remarkable among all types of PDI discotic liquid crystals.

**Figure 13:**
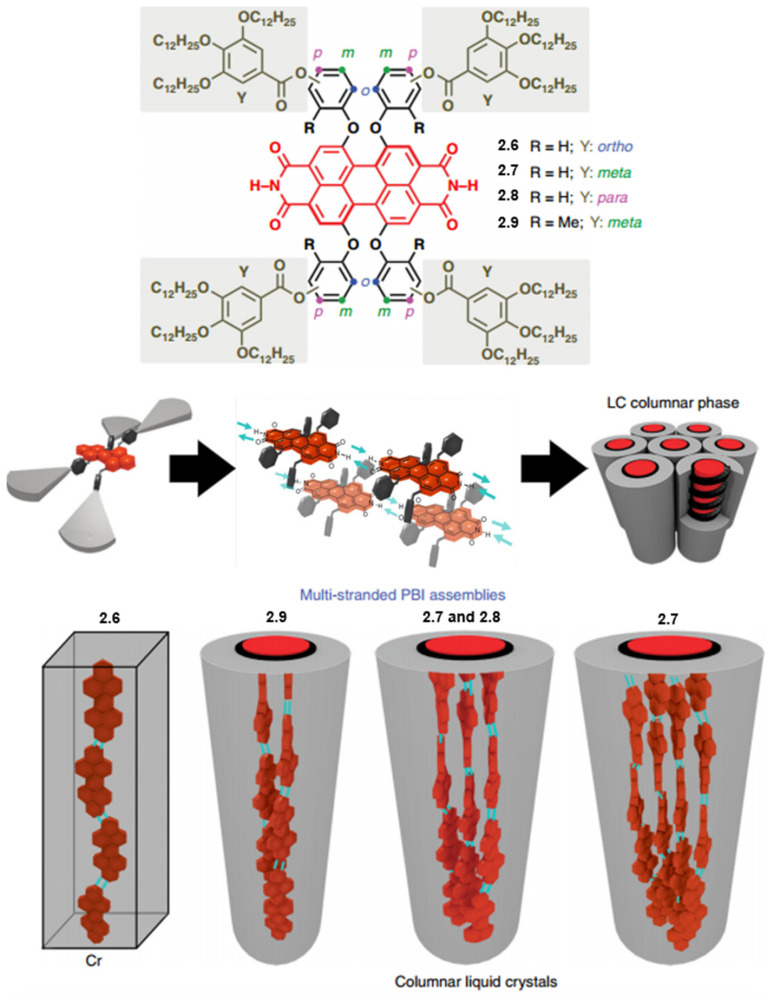
Molecular structures of PDI derivatives **2.6**–**2.9** and their corresponding schematic representations of self-assembly mechanisms [78].

Würthner et al. report a series of crystalline and LC PDIs self-assembling into single or multi-stranded (two, three, and four strands) aggregates with predominant J-type exciton coupling as shown in Figure 13 [78]. The present series of compounds (PDIs **2.6**–**2.9**) was designed by using four different phenoxy spacers (ortho-, meta-, para-hydroxyl-, and 2-hydroxy-6-methyl phenoxy) and 3,4,5-tridodecyloxybenzoate dendrons. All PDI derivatives self-assemble in ordered, anisotropic structures independent of the substitution pattern at the phenoxy spacers. PDI **2.6** forms a crystalline phase while PDIs **2.7**–**2.9** generate LC phases. Meanwhile, PDI derivatives **2.7**–**2.9** formed strongly coupled J-aggregates, whereas PDI derivative **2.6** exhibited only weak coupling between transition dipole moments. This demonstrates how the molecular engineering of PDI dyes can be utilized to design complex supramolecular assemblies with unprecedented stacking modes and intriguing optical properties. A tetraphenoxy dendronized PBI with two N-H functional groups has been shown to form H-bonded linear chains with slipped stacking arrangements of twisted PDI cores. This results in LC and crystalline columnar J-aggregates where the chromophores are aligned parallel to the columnar axis. Remarkably, the composition of these columnar assemblies can be tuned to consist of one, two, three, or four chains, depending on the molecular design imparted by the appropriate dendritic wedge. In this context, the relative position of the dendritic moieties at the phenoxy spacers of the PDI core determines the spatial requirements (i.e., the twist of the perylene core, the pitch, and consequently, the number of strands in a single column). Moreover, these structural features directly translate into functional properties due to the electronic coupling of the transition dipole moments of tightly stacked dyes. Here, it is demonstrated that the red-shift of the absorption maximum in the aggregated state, compared to the monomer, depends on the number of self-assembled chains and provides optimal J-aggregation (with purely negative coupling between adjacent molecules) for the double-chain arrangement of PDI **2.9**. Strongly coupled J-aggregates are promising materials for photonic devices, with significant breakthroughs in the study of optical microcavities.

## 3. AIE-Modified PDI-Based Discotic Liquid Crystals

**Figure 14:**
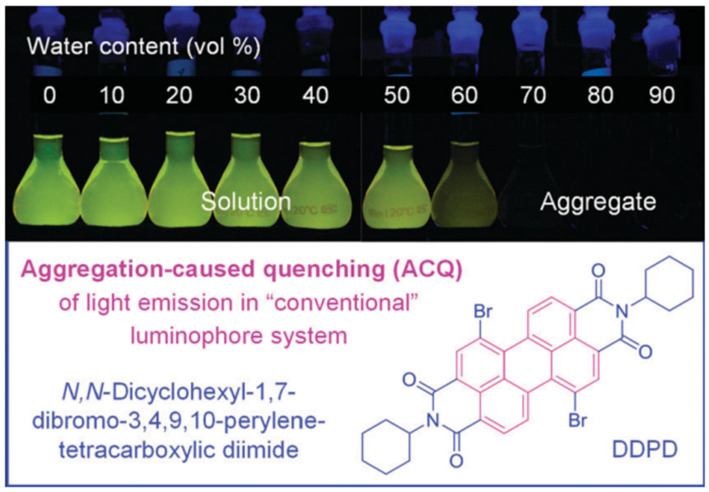
Fluorescence images of DDPD (10 mM) solutions/suspensions in THF/water mixtures with varying water contents [79].

Perylene diimide is a well-established lumophore consisting of multiple fused planar aromatic rings, which enhances the extent of π-π coupling by increasing the conjugation length and promoting stronger electronic interactions between the aromatic units [79,80,81]. The resulting larger discotic stacking can indeed lead to more efficient light emission in solution, but at the same time, their ACQ effect becomes more severe [82,83]. As depicted in Figure 14, N, N-Dicyclohexyl-1,7-dibromo-3,4,9,10-perylene-tetracarboxyldiimide (DDPD) is not only a typical discotic liquid crystal (DLC) molecule but also an excellent lumophore. As an example of the ACQ effect, DDPD shows high luminescence intensity in diluted THF solution, but its emission significantly decreases when water is added to the THF, as the immiscibility of DDPD with water increases the local concentration of lumophore, leading to the aggregation of DDPD molecules [84,85,86]. As the water content increases to 60 vol%, the solvation ability of the THF/water mixture diminishes, causing most DDPD molecules to aggregate. The formation of these aggregates results in the complete quenching of DDPD luminescence. The central disk-shaped perylene core of the DDPD molecule likely undergoes strong π-π stacking interactions within the aggregate, contributing to the observed quenching [87,88].

In 2001, Benzhong Tang’s research group observed an intriguing phenomenon while investigating silicon-based hole compounds, such as 1-methyl-1,2,3,4,5-pentaphenylsilicone [89]. The compound exhibited no light emission when dissolved but emitted light upon aggregation, a behavior that contrasts with the typical ACQ effect observed in many conventional organic fluorescent materials [90,91]. Subsequently, Tang and colleagues measured the fluorescence quantum yield (FF) of this compound in an ethanol/water system and discovered that its FF reached 0.21 when the water fraction was 90%, representing a 333-fold increase compared to its value in ethanol solution. This result demonstrated the compound’s enhanced fluorescence emission upon aggregation [92]. As this light emission is triggered by aggregation, Tang and colleagues coined the term “aggregation-induced emission” (AIE) to describe this phenomenon [93,94]. By modifying these systems with specific substituents, it is possible to mitigate ACQ and enhance fluorescence through AIE. Since the introduction of the concept of AIE, numerous research groups have focused on designing and synthesizing new AIE luminophores, optimizing and modulating their aggregated forms, and investigating and controlling their emission behaviors. A significant number of AIE-active molecules have been synthesized [95]. One such molecule is hexaphenylsilane (HPS), a helical, non-planar compound. The unique structural characteristics of HPS give rise to distinct emission behaviors. In dilute solution, the six phenyl rotors of the HPS molecule undergo dynamic rotation around the silicon core, which results in the non-radiative deactivation of the excited state, effectively preventing the molecule from emitting light [96,97]. In aggregates, HPS molecules cannot stack through the π-π stacking process due to their helical shape, and their phenyl rotors’ molecular rotation is significantly restricted due to physical constraints [31]. This restriction on intramolecular rotation (RIR) blocks the non-radiative pathways and opens up the radiative channel. Therefore, HPS molecules emit light in the aggregated state. The competition between AIE and ACQ, two completely opposite luminescent properties, has attracted the interest of researchers [98,99,100].

**Figure 15:**
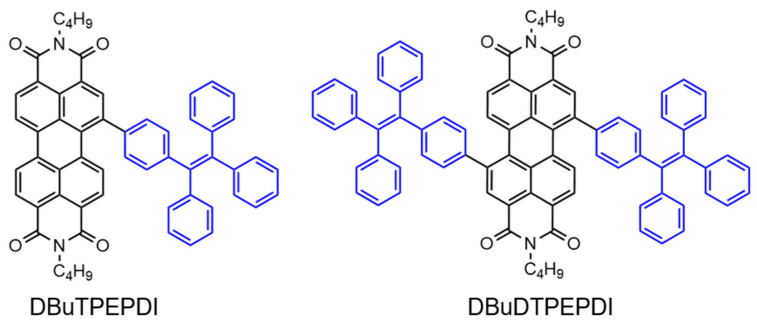
Molecular structures of the mono- and di-TPE-substituted PBIs (The black part represents the core structure of the perylene diimide molecule, while the blue part corresponds to the AIE molecule substituent group) [105].

Since Tang and his colleagues first reported the phenomenon of AIE [89], extensive research has been conducted on the development of AIE-active optoelectronic materials and their applications across various optoelectronic fields [89]. Notably, the modification of PDI-based discotic liquid crystal molecules has attracted significant attention from researchers due to their promising properties and potential for diverse applications [101,102,103,104]. In 2012, Tang’s group chemically modified PDIs by attaching two tetraphenylethylene (TPE) groups to its bay positions as represented in Figure 15. This modification resulted in a significant fluorescence red-shift (over 120 nm) and endowed the derivatives (1,6-/1,7-dtpePDIs) with distinct AIE behavior [104,105]. Both 1,6-dtpePDIs and 1,7-dtpePDIs exhibited bright red fluorescence in the solid state, marking the successful transition of PBI from the traditional ACQ behavior to AIE behavior. This transition demonstrated that modifying ACQ molecules with TPE groups is an effective strategy for constructing novel AIE-active materials. The FF of 1,7-dtpePDIs aggregates (solid-state FF = 29.7%, liquid-state FF = 90% when formed in hexane/dichloromethane mixtures) was approximately 424 times higher than that observed in dichloromethane solution (FF = 0.07%). Electrochemical studies confirmed that 1,7-dtpePDIs retained the inherent n-type semiconductor properties of the PBI core. In various nonsolvent/solvent mixtures (e.g., water/THF, methanol/DCM, hexane/DCM, methanol/dioxane), the bulky and twisted TPE moieties attached to the PBI core did not impede the formation of ordered assemblies. Well-defined one-dimensional (1D) microstructures, including fibers, wires, and rods, were obtained, which emitted bright red fluorescence and demonstrated waveguiding properties [106,107,108].

Subsequently, Tang and colleagues investigated the effect of substituent numbers on the AIE performance of PDIs by comparing the fluorescence properties of unsubstituted, mono-, and di-TPE-substituted PDIs. The results showed that DBuT-PEPDIs exhibited weak characteristic PBI emission in solution. In contrast, solid-state fluorescence studies demonstrated that TPE-modified PBIs exhibited typical AIE behavior. This finding highlighted that the fluorescence (FL) properties of PBIs could shift from ACQ to AIE with the modification of a single TPE fragment. However, attaching only one TPE group to the larger PBI core partially altered its fluorescence characteristics. In dilute solution, the mono-TPE-substituted PBI (DBuTPEPDIs) displayed characteristic fluorescence features, albeit with a reduced FF (2.2%). When aggregates were formed in hexane/DCM mixtures (fh = 90%), the powder samples emitted red fluorescence. In contrast, the di-TPE-substituted PDIs (DBuDTPEPDIs) exhibited a very low FF (0.07%) and poor solubility in dilute solution. However, in the aggregated state, formed in hexane/DCM mixtures (fh = 90%), these molecules emitted efficient red-to-near-infrared FL in the powder form.

**Figure 16:**
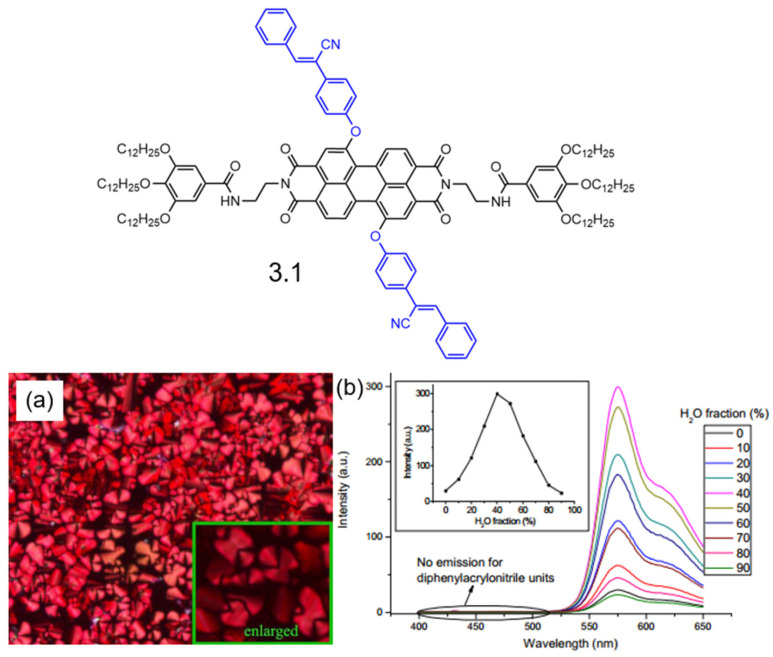
Molecular structures of PDI derivative **3.1**: (**a**) POM texture of PDI derivative **3.1** obtained upon cooling at 150 °C, with magnification at 200× for the large image and 500× for the smaller image. (**b**) Emission spectra of compound **6** in THF/H_2_O mixtures with varying water fractions (2 × 10^−6^ M) excited at λ = 330 nm. (Inset: variation in fluorescence intensity as a function of water fraction in THF/H_2_O mixtures) [109].

In 2017, Yang et al. successfully synthesized a 1,7-disubstituted PDI derivative (**3.1**), incorporating the AIE-active molecule diphenylacrylonitrile, with a high yield as indicated in Figure 16 [109]. This highly substituted PDI derivative exhibited well-ordered hexagonal columnar liquid crystal behavior within the temperature range of 124.9–189.5 °C. Upon excitation at 480 nm, the fluorescence intensity decreased as the water content in THF/H_2_O mixtures increased. However, when excited at 330 nm, a significant fluorescence enhancement was observed, with the intensity reaching its peak at a water fraction of 40%. This resulted in a tenfold increase in fluorescence intensity and a pseudo-Stokes shift of 251 nm. The observed phenomena were successfully explained by AIE and Förster resonance energy transfer (FRET) effects, which were further corroborated by fluorescence quantum yield and lifetime studies.

**Figure 17:**
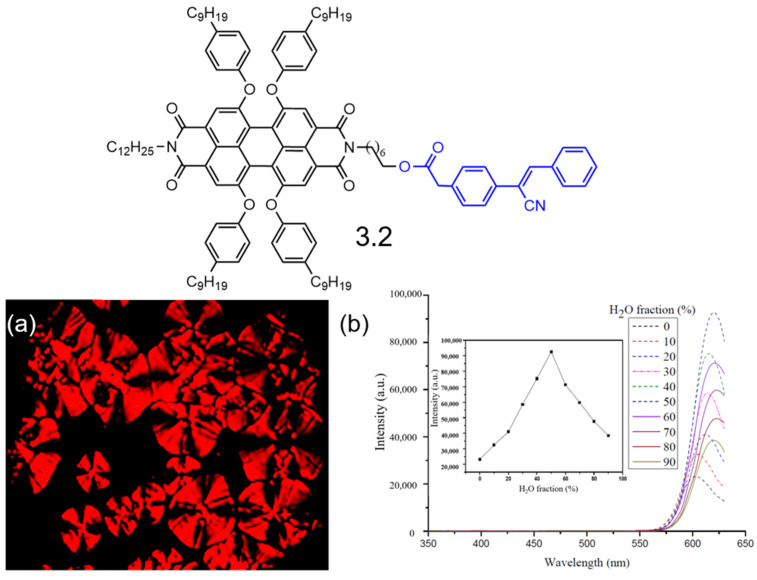
Molecular structures of PDI derivative **3.2** (The black part represents the core structure of the perylene diimide molecule, while the blue part corresponds to the AIE molecule substituent group): (**a**) POM image of PDI derivative **3.2** in the mesophase at 120 °C. (**b**) Emission spectra of PBI 5 in a 10 mM THF–water system with varying water fractions (f_w_), excited at λ_ex_ = 330 nm. (Inset: variation in fluorescence intensity as a function of water fraction, f_w_) [110].

In 2018, the Yang group successfully incorporated diphenylacrylonitrile into the side chains of the amine substituents on PDIs, resulting in the synthesis of PDI derivative **3.2** as described in Figure 17 [110]. DSC, POM, and XRD studies confirmed that this material could self-assemble into a stable hexagonal columnar liquid crystal phase within the temperature range of 56–160 °C. The PDI derivative also exhibited robust fluorescence in solution, thin films, and mesophases, which can be attributed to the synergistic effects of AIE and FRET between the diphenylacrylonitrile and perylene moieties. It achieved a pseudo-Stokes shift of 283 nm, with fluorescence quantum yields ranging from 0.62 to 0.79 in solution and from 0.68 to 0.86 in the solid state. This study presents an effective strategy for converting columnar liquid crystals with ACQ effects into those exhibiting AIE effects, successfully bridging the gap between outstanding columnar mesomorphic properties and strong solid-state fluorescence. The structural features of TPE and diphenylacrylonitrile play critical roles in inducing AIE in PDIs-DLCs. For TPE, the steric hindrance created by its phenyl groups limits molecular motion and prevents the formation of tightly packed aggregates prone to ACQ. This loose packing promotes efficient radiative transitions in the aggregated state. Diphenylacrylonitrile, on the other hand, contributes through its electron-rich and electron-deficient regions, which enhance electronic coupling with the PDI core and allow for extended π-conjugation in the aggregated state. These features collectively result in fluorescence enhancement and make these systems highly suitable for solid-state optoelectronic devices.

These findings suggest that for smaller conjugated systems, the attachment of a single TPE unit to the core is sufficient to transition the emission behavior from ACQ to AIE.

## 4. Discotic Liquid Crystal-Modified PDI Derivatives

DLCs, first discovered by Chandrasekhar in 1977, typically feature a planar aromatic core surrounded by multiple peripheral flexible chains [111,112,113]. In 1994, D. Haarer reported that hexahexylthio-triphenylene (HHTT) demonstrated high hole charge carrier mobility of up to 0.1 cm^2^ V^−1^ s^−1^ comparable to that of amorphous silicon. This significant finding sparked considerable interest in triphenylene-based DLCs due to their potential for electronic applications [114,115]. Triphenylene derivatives offer several advantages, including tunable chemical structures, ease of processing, self-assembly capabilities, and relatively high charge carrier mobility. Furthermore, their electron-rich nature contributes to excellent hole transport properties, making them highly suitable for various electronic applications [31,116,117,118]. The design and fabrication of triphenylene and perylene-based donor–acceptor (D-A) bipolar transport self-organizing semiconductor structures have garnered significant interest from researchers. These structures facilitate the spontaneous nanoscale segregation of p-type and n-type domains within the bulk material, thereby creating well-defined and distinct conductive pathways for each type of charge carrier. This self-organization enhances charge transport efficiency and holds great promise for advanced electronic applications [119,120,121]. Investigating the relationship between the self-assembly of D-A liquid crystals with nanoscale segregated structures and their charge transport properties offers valuable insights into the fundamental mechanisms that govern charge dynamics. This understanding can serve as a foundation for the rational design of high-performance liquid crystal materials, particularly in the field of OPVs, where optimized charge transport is crucial for device efficiency [122,123].

**Figure 18:**
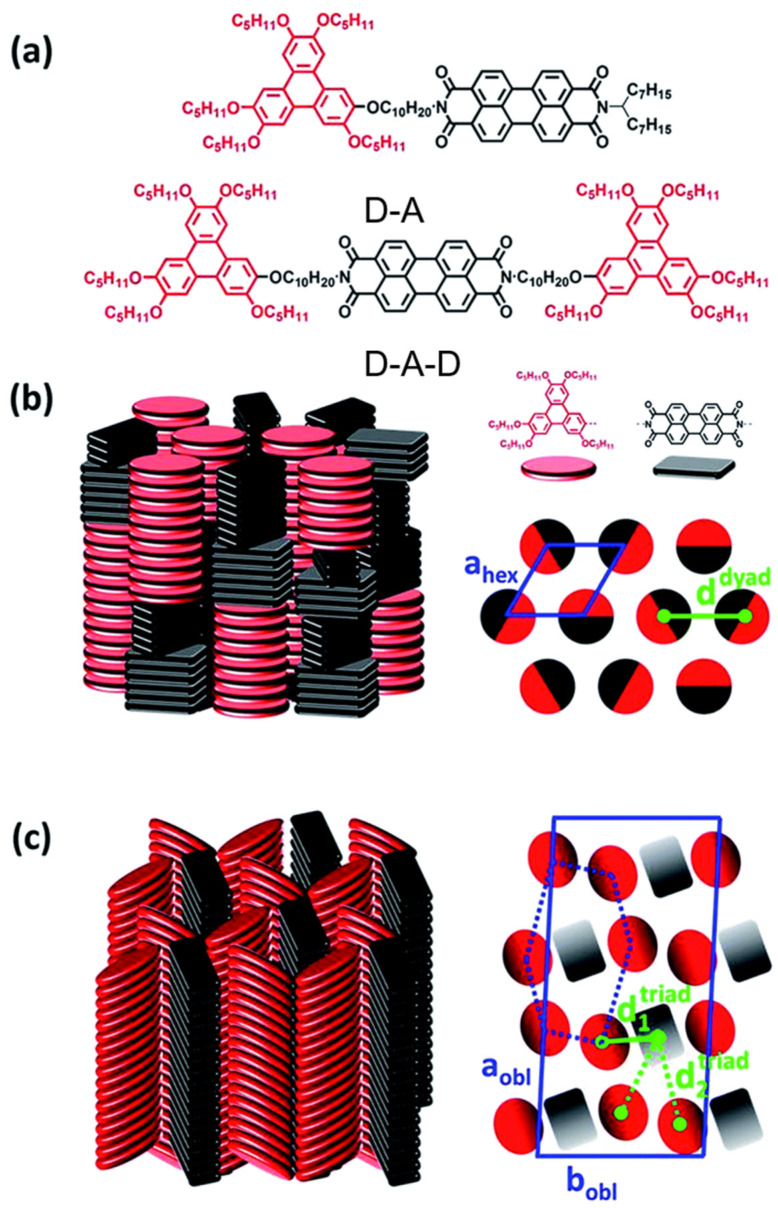
(**a**) Chemical structures of the dyad and triad molecules. (**b**) A schematic representation of (**left**) the self-organization within the Col_hex_ mesophases of the D–A dyad and (**right**) the hexagonal lattice (blue lozenge) formed by undifferentiated columns. The distance d^dyad^ ≈1.9 nm (green line) corresponds to the average center-to-center spacing distance between D and A columns. (**c**) A schematic representation of (**left**) the self-organization within the Col_obl_ mesophases of the D–A–D triad after annealing and (**right**) the oblique lattice (blue parallelogram) formed by intermingled distinct columns located at the nodes of distorted hexagonal lattices (blue dashed distorted hexagon). The distances d_1_^triad^ ≈ 1.4 and d_2_^triad^ ≈ 2.5 nm (respectively green solid and dashed line) correspond to the two average center-to-center spacing distances between D and A columns coexisting within the triad columnar arrangement [124].

Ribierre et al. designed and synthesized two self-assembled discotic liquid crystalline D–A and donor–acceptor–donor (D–A–D) systems, incorporating triphenylene and PDI units as shown in Figure 18. These systems were engineered to explore the interplay between their molecular structure and self-assembly behavior, aiming to optimize charge transport and light absorption properties for potential applications in organic electronics [124]. The photophysical properties of self-assembled discotic liquid crystalline D–A and D–A–D systems, composed of triphenylene and PDI units, were investigated in thin films using steady-state optical spectroscopy and subpicosecond transient absorption measurements. These systems, in which triphenylene and PDI units are covalently linked by flexible decyloxy chains, serve as the electron donor (D) and acceptor (A), respectively. The liquid crystalline structures formed exhibit well-separated donor and acceptor π-stacked columnar arrangements in thin films. In the dyad and triad systems, the molecules spontaneously self-assemble into columnar nanostructures with distinct arrangements of D and A units. Dyads form alternate D–A stacks, while triads display more complex interwoven structures. Absorption spectra of the films indicate the aggregation of the PDIs and triphenylene moieties along the columns. Steady-state photoluminescence measurements show significant fluorescence quenching, primarily due to photoinduced charge transfer (CT) processes occurring between the triphenylene donor and PDI acceptor units. Subpicosecond transient absorption measurements reveal that photoinduced CT states in the dyad and triad films form within 0.3 ps and recombine on a time scale of 150–360 ps. A correlation between charge recombination dynamics and the spacing between donor and acceptor units was observed, with longer D–A spacing extending the lifetime of CT states without significantly affecting their formation rate. This finding suggests a viable strategy for optimizing molecular design in these systems. The flexible alkyl chains connecting the D and A units allow for tunable self-assembly and enhanced optoelectronic properties. This study provides valuable insights into how molecular packing influences charge transfer dynamics and lays the groundwork for the rational design of high-performance organic electronic materials.

**Figure 19:**
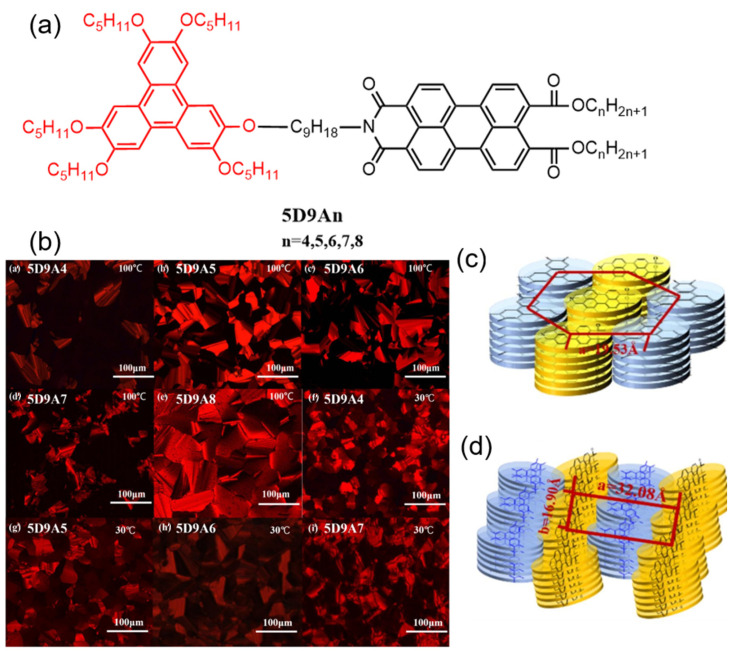
(**a**) Molecular structures of the 5D9An series of compounds (The black part represents the core structure of the perylene diimide molecule, while the red part corresponds to the substituent groups of the disc-shaped liquid crystal molecules); (**b**) POM images of all dyads cooling from the isotropic liquid at 0.5 °C min^−1^ ((**a′**) 5D9A4 in 100 °C; (**b′**) 5D9A5 in 100 °C; (**c′**) 5D9A6 in 100 °C; (**d′**) 5D9A7 in 100 °C; (**e′**) 5D9A8 in 100 °C; (**f′**) 5D9A4 in 30 °C; (**g′**) 5D9A5 in 30 °C; (**h′**) 5D9A6 in 30 °C; (**i′**) 5D9A7 in 30 °C); (**c**) Colh molecular stack diagram of 5D9A8; (**d**) Colr molecular stack diagram of 5D9A4 [125].

Zhang’s research group synthesized a series of D–A liquid crystals, incorporating pentoxyl benzofluorene as the electron donor, PDI units as the electron acceptor, and nonylamine as the bridging chain [125,126]. It was found that these D–A liquid crystals form highly ordered rectangular or hexagonal columnar phases at lower temperatures while transitioning to hexagonal columnar phases at higher temperatures. Specifically, 5D9A8 and 5D9A4 formed nano-segregated structures, where the D–A components self-assembled into separate columns, exhibiting distinct hexagonal and rectangular columnar phases, as shown in Figure 19c,d, respectively. Characterization using steady-state spectroscopy revealed that in the solid state, the benzofluorene and perylene cores stack separately into columns, forming a well-defined nano-segregated structure. Notably, this structure demonstrated charge carrier mobility up to 10^−3^ cm^2^V^−1^s^−1^, highlighting that the D-A segregated arrangement creates distinct channels for hole and electron transport, effectively enhancing charge carrier mobility.

## 5. Summary and Future Outlook

Perylene diimide-based liquid crystals (PDIs-DLCs) represent a highly promising class of materials with significant potential for a wide range of advanced applications. The imide and bay positions on the PDI core offer extensive opportunities for functionalization, enabling the nearly unlimited customization of these molecules. In addition to conventional alkyl chains and basic substituents, a diverse array of specialized functional groups can be incorporated into PDI molecules, leading to multifunctional materials with a broad spectrum of exceptional properties. Integrating AIE molecules into perylene-based discotic liquid crystal molecules is a promising approach with potential applications and can address the challenges posed by the ACQ effect. Advanced molecular design strategies are needed to enhance charge transport properties. However, research on this integration remains relatively sparse. The development of more complex substituents and advanced modification methods presents significant obstacles. Achieving the effective integration of PDIs-DLCs with high-performance molecules remains a critical challenge, and advancing this area is essential for realizing the full potential of these materials in practical applications.

To contextualize the strengths and weaknesses of PDI-based DLCs in organic electronics, they exhibit high electron mobilities, often surpassing traditional n-type semiconductors like fullerene derivatives. However, their hole mobilities are lower compared to p-type materials like pentacene and rubrene due to their n-type nature. Their fluorescence quantum yields and absorption spectra are competitive, especially in applications like OPVs. While the synthesis of PDIs-DLCs, particularly with complex functionalization, poses scalability challenges, their molecular precision and thermal stability offer advantages for high-performance applications. Additionally, their discotic liquid crystalline nature enables the formation of highly ordered thin films, crucial for optoelectronic devices requiring molecular alignment, unlike amorphous organic semiconductors that, while easier to process, lack self-organizing capabilities.

In-depth research and the exploration of novel PDIs-DLC molecules possess considerable practical significance and offer substantial potential for diverse applications across multiple fields. These include OFETs, fluorescent solar concentrators, electrophotographic devices, dye lasers, and OPVs. By addressing challenges in scalability, stability, device integration, manufacturing, cost-effectiveness, and commercial viability, PDIs-DLCs can achieve breakthroughs in performance and functionality, broadening their applicability and impact in both scientific research and industrial sectors.

## Data Availability

Not applicable.
